# Visualized Exploratory Spatiotemporal Analysis of Hand-Foot-Mouth Disease in Southern China

**DOI:** 10.1371/journal.pone.0143411

**Published:** 2015-11-25

**Authors:** Ji-Xia Huang, Jin-Feng Wang, Zhong-Jie Li, Yan Wang, Sheng-Jie Lai, Wei-Zhong Yang

**Affiliations:** 1 College of Forestry, Beijing Forestry University, Beijing, China; 2 State Key Laboratory of Remote Sensing Science, Beijing Normal University, Beijing, China; 3 State Key Laboratory of Resources and Environmental Information System, Chinese Academy of Sciences, Beijing, China; 4 Key Laboratory of Infectious Disease Surveillance and Early-Warning, Chinese Center for Disease Control and Prevention, Beijing, China; 5 Beijing Research Institute of Water Affair Automation, Beijing, China; Shanxi University, CHINA

## Abstract

**Objectives:**

In epidemiological research, major studies have focused on theoretical models; however, few methods of visual analysis have been used to display the patterns of disease distribution.

**Design:**

For this study, a method combining the space-time cube (STC) with space-time scan statistics (STSS) was used to analyze the pattern of incidence of hand-foot-mouth disease (HFMD) in Guangdong Province from May 2008 to March 2009. In this research, STC was used to display the spatiotemporal pattern of incidence of HFMD, and STSS were used to detect the local aggregations of the disease.

**Setting:**

The hand-foot-mouth disease data were obtained from Guangdong Province from May 2008 to March 2009, with a total of 68,130 cases.

**Results:**

The STC analysis revealed a differential pattern of HFMD incidence among different months and cities and also showed that the population density and average precipitation are correlated with the incidence of HFMD. The STSS analysis revealed that the most likely aggregation includes the Shenzhen, Foshan and Dongguan populations, which are the most developed regions in Guangdong Province.

**Conclusion:**

*Both STC and STSS are efficient tools for the exploratory data analysis* of disease transmission. STC clearly displays the spatiotemporal patterns of disease. Using the maximum likelihood ratio, the STSS model precisely locates the most likely aggregation.

## Background

In epidemiological research, we want to detect the aggregation of a disease, and the effective visualization of the distribution of a disease is important to this end. To date, various theoretical models have been established to detect disease outbreaks in time or space [1–11]. Although precise local aggregations are obtained using these analytical, theoretical models, the results of these models are abstract and not easily represented. The use of visual analysis technologies in epidemiological studies can contribute to an understanding of the distribution of disease. Currently, most data in epidemiology are spatiotemporal data, and the visualization of spatial and temporal information is an important problem.

Some visual analysis methods have been developed to display data concerning disease distribution [12–17]. Parallel coordinates [15] can be used to visualize multi-dimensional data, but this method exhibits several shortcomings when expressing spatial relationships. Self-organizing maps (SOM) [16] are good visual tools for the analysis of clustering in multi-dimensional data, but this method cannot sufficiently represent the spatial and temporal properties. The Star map visualization technology [17] displays discrepancies in spatial properties, but this analysis needs to improve for the visualization effect in the time dimension. In the early 1970s, Hägerstrand developed a model called the “space-time cube” [18]. In this model, he used the base of a cube to denote the geographic space and the vertical axis to represent the temporal dimension. Thus, the changes in the spatial patterns in the geography could be represented by the vertical axis. Each frame, which reflects the spatial pattern at a particular time, could be obtained by slicing a particular location in the time dimension. The “space-time cube” model had been used in various fields [19], and the experimental results have demonstrated that the “space-time cube” could be effectively applied to geography, the environment and other domains.

This study changed the coordinate layouts in the traditional space-time cube (STC). In the traditional STC, the cube’s base represents the geography (along the x- and y- axes), while the cube’s height denotes time (z-axis) [20]. However, in our research, we used the x- and y-axes to represent the month and day, respectively, and the z-axis to denote geographical space. The reason for this coordinate layout was to facilitate the observation of the total incidence of disease in a given location or time.

In recent years, HFMD has rampantly spread throughout the western region of the Pacific Ocean, including China, Japan, Singapore, and Malaysia [21–23]. The incidence of HFMD, particularly among children, is increasing in these regions [24]. Every year, a large number of children are infected with HFMD. Thus, it is necessary to study the spatiotemporal pattern of HFMD to prevent the further spread of this disease.

This study combines the methods of the space-time cube [18] and space-time scan statistics [4] to analyze the spatiotemporal pattern of HFMD incidence in Guangdong, a southern province in China. The space-time scan statistic method is used for geographical disease surveillance as well as for the evaluation of geographical disease cluster alarms [25]. The spatiotemporal distribution of this disease could be visualized through the space-time cube, and disease aggregation could be achieved using space-time scan statistics. The remaining sections of this paper are organized in the following manner: the hand-foot-mouth disease data that was obtained from May 2008 to March 2009 in Guangdong Province are provided in section DATA. In section METHODS, we introduce the experimental method that was used in this research, combining the space-time cube with space-time scan statistics. Section RESULTS presents the results of the visual analysis and scan statistics, with a discussion about interesting information concerning HFMD in Guangdong as obtained from these results. Finally, we summarize our work, discuss the advantages and disadvantages of this method, and provide a description of future directions in Section DISCUSSION.

## Data

**Fig 1:**
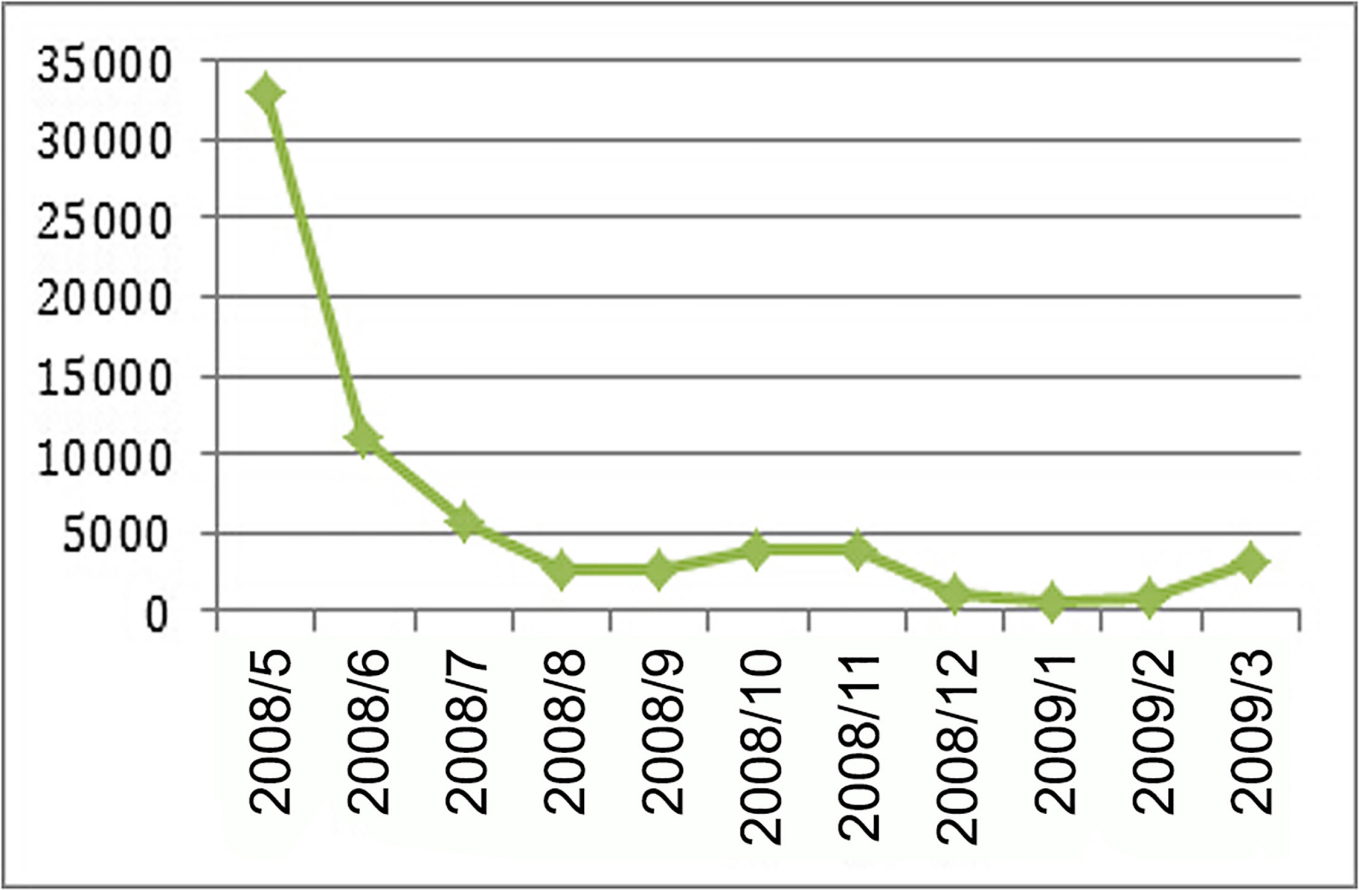
HFMD case statistics from May 2008 to March 2009 in Guangdong Province.

The HFMD dataset that was used in this research was collected at the Chinese Center for Disease Control and Prevention (CDC) from May 1, 2008, to March 27, 2009, in Guangdong Province. Located in the southern region of China, Guangdong Province is China’s most economically developed region and has the largest population density. In recent years, the incidence of HFMD among children under 5 years of age has increased in Guangdong Province. The total number of HFMD cases in our study was 68,130. Fig 1 illustrates the number of HFMD cases from May 2008 to March 2009. The number of HFMD cases decreased over this time period, with most cases occurring in May and June 2008.

In our research, the minimum geographical unit is a prefecture-level city containing a total of 21 cities in Guangdong Province; the time resolution is one day; and the number of days is 331. The demographic characteristics of the prefecture-level cities are summarized in Table 1. As the incidence of HFMD was primarily detected among children, the calculation of HFMD incidence was based on HFMD cases in children aged 0–9 years as the numerator, and the total population aged 0–9 years was used as the denominator [26]. Population data were collected from the China Statistical Yearbook for Cities [27], and precipitation data were collected from the China Meteorological Data Sharing Service System.

**Table 1:** Demographic characteristics of the prefecture-level cities of Guangdong Province.

CODE	City	East longitude	Northlatitude	Area(*km* ^2^)	Population(10,000 person)	GDP(10000 Yuan)
4401	Guangzhou	113.54	23.37	7434	784.23	82,158,151
4402	Shaoguan	113.77	24.82	18,493	322.78	5,252,835
4403	Shenzhen	114.14	22.66	1953	228.07	78,065,387
4404	Zhuhai	113.31	22.21	1701	99.48	9,920,616
4405	Shantou	116.55	23.33	2068	506.60	9,747,835
4406	Foshan	112.95	23.01	3848	364.34	43,333,044
4407	Jiangmen	112.67	22.30	9554	389.78	11,720,185
4408	Zhangjiang	110.16	21.10	12,941	727.36	10,405,935
4409	Maoming	110.95	22.02	11,458	725.81	10,388,505
4412	Zhaoqing	112.20	23.54	15,230	420.27	7,639,416
4413	Huizhou	114.50	23.25	11,195	318.75	11,696,057
4414	Meizhou	116.08	24.20	15,899	504.49	5,299,749
4415	Shanwei	115.52	23.02	4831	337.82	2,691,674
4416	Heyuan	114.96	24.04	15,808	344.44	4,849,781
4417	Yangjiang	111.77	22.04	8091	273.21	4,839,429
4418	Qingyuan	112.87	24.31	19,194	405.74	8,000,265
4419	Dongguan	113.88	22.94	2465	174.87	37,025,344
4420	Zhongshan	113.38	22.52	1800	146.43	14,085,194
4451	Chaozhou	116.77	23.80	3113	256.16	4,428,755
4452	Jieyang	116.12	23.34	5284	641.21	7,102,884
4453	Yunfu	111.80	22.82	7980	272.72	2,912,282

## Methods

### Space-Time Cube

Generally, spatiotemporal data include three main components: Space (where), Time (When) and Attribute (What) [28–29]. Although these components could be represented using a map, time sequence or STC model, their differences are apparent. Both space and attribute information can be represented using a static map, but it is difficult to reflect the change in the attribute across the time dimension. A time series can be used to reflect the trend of an attribute across the time dimension, particularly when the attribute exhibits a specific trend. However, it is difficult to observe the distribution of the data in the space dimension. Compared with the pure map and time series, the STC model has many advantages. The STC model could simultaneously reflect the changes in attribute information in both the time and space dimensions. Therefore, we used the STC model to characterize the changes in the spatiotemporal patterns of HFMD incidence.

**Fig 2:**
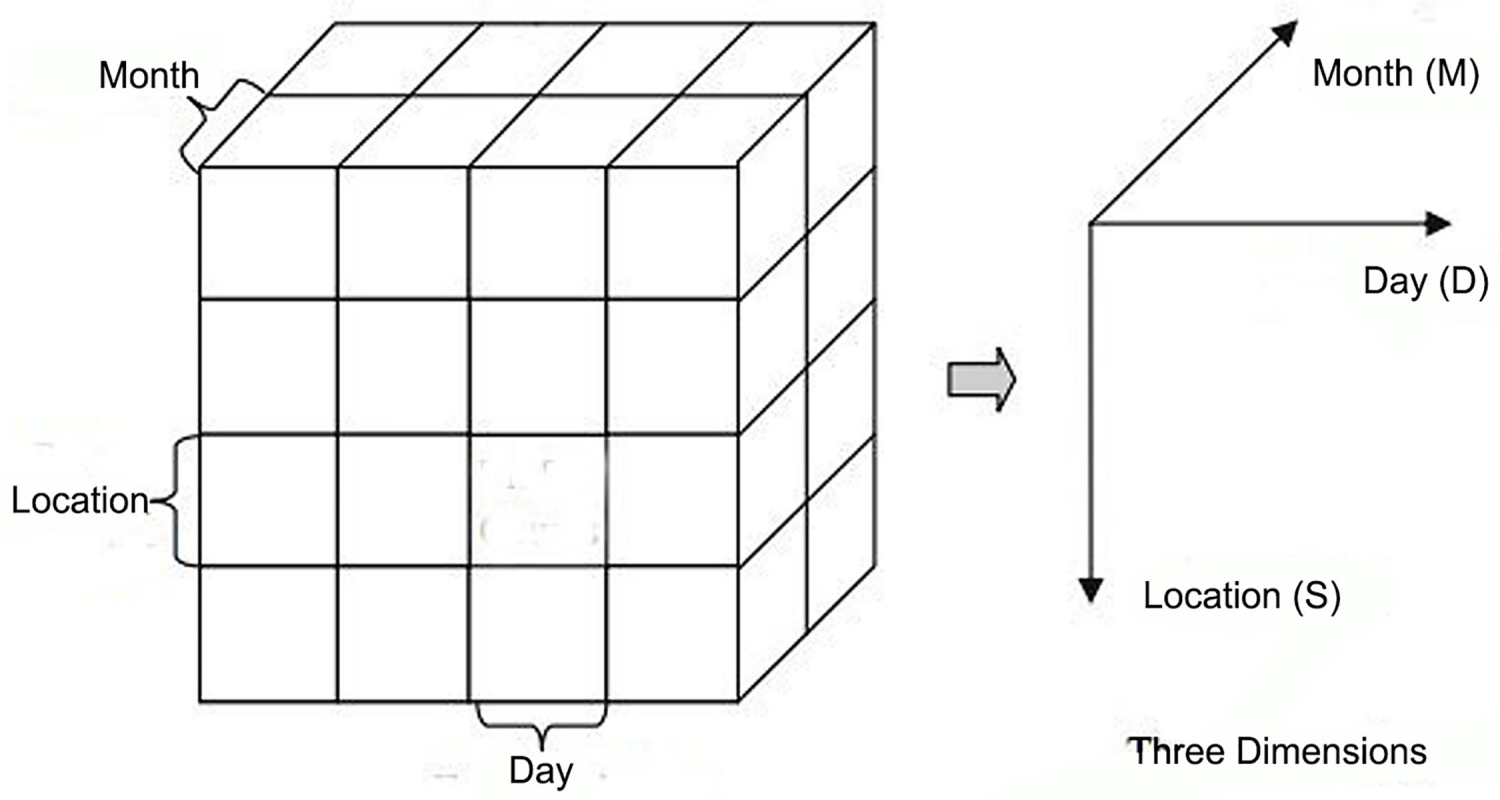
A spatiotemporal cube as defined with three dimensions [31]

The coordinate layout that was used in this study was different from the traditional space-time cube. We used one horizontal axis to represent the month, another horizontal axis to denote the day, and the vertical axis to display the geographical space (Fig 2). The reason for this coordinate layout was to facilitate the observation of the total HFMD incidence in a given time or space. Along the vertical axis, geographical units could be arranged according to the environmental factors; through this arrangement, the impact between these factors and the incidence of HFMD could be observed. For example, if the geographical units are arranged in accordance with the population density, we could observe whether the population density impacts the incidence of HFMD; if the geographical units are arranged according to the average temperature, then the impact of the average temperature on the incidence of HFMD could be observed.

We observed the incidence of HFMD from the visible faces of the space-time cube (Fig 2). For example, on the front face where the month dimension is fixed, the entire phenomenon occurring in a particular month could be observed from the plane. Every face is divided into crisscross grids, and the color of each grid represents the incidence of HFMD. We use red to denote high incidence and blue to denote low incidence. The operations of the space-time cube include slice, dice, drill-down, roll-up and pivot [30].

### Space-Time Scan Statistics

Kulldorff proposed the spatial scan statistic model in 1997. In this study, Kulldorff used a series of variable scanning windows to detect likely clusters in space [3]. Subsequently, to evaluate space-time cluster alarms, Kulldorff proposed space-time scan statistics and used brain cancer cases in Los Alamos to confirm this model [4]. The software SaTScan facilitates the application of these methods [32–33].

The entire research region G is divided into a series of sub-regions {*z*_*i*_ | *i* = 1, 2, …, *k*}. There are no gaps between these sub-regions, and each sub-region is not overlapped or intersected by other sub-regions. To calculate the relative risk coefficients (*p*_*i*_) of the disease in all of the sub-regions, this model assumes null and alternative hypotheses. The null hypothesis is that the relative risk coefficients (*p*_*i*_) of the disease in all of the sub-regions are identical. The alternative hypothesis is that there is always an aggregation; within the aggregation, the relative risk coefficients (*p*_*i*_) of the disease are identical; and outside of the aggregation, the relative risk coefficients (*q*_*i*_) of the disease are also identical, while *p*_*i*_ > *q*_*i*_ [25].

In space-time permutation scan statistic model, the scanning windows were defined as thousands or millions of overlapping cylinders, each being a possible candidate for an outbreak [32]. The base of the cylinder denotes the geographical space of the potential outbreak. Generally, we first iterate over a finite number geographical grid points and then gradually increase the radius of circle from zero to some maximum value defined by the user [32]. The height of the cylinder denotes the number of days. If cases obey a certain distribution (e.g., Poisson distribution), then the disease cases and population inside and outside of the cylinder are obtained, together with the expected number of cases and the population at risk. For each cylinder, the likelihood is calculated using these numbers. The cylinder, maximum likelihood, and expected number of cases represent the most likely cluster [3].

### Building the HFMD database

To facilitate the visualization and analysis of the data, the data format should be normalized. In this study, we constructed the HFMD database with three sub-databases. Although the database construction might appear to be application-specific, the properties in disease data are similar. Therefore, these sub-datasets are typically used for other disease studies, and even for other types of data.

The first dataset is prepared for the visualization of the space-time cube and includes 35 fields (Table 2). The field CODE represents the administrative code of the prefecture-level city, which is usually a six-digit number in China. The field NAME shows the name of the prefecture-level city, and the field POPU denotes the population aged 0–9 years in each prefecture-level city. The field MONTH denotes the month when the HFMD data were collected. The remaining fields from DAY1 to DAY31 denote the number of HFMD cases from the first day to the last day of each month. If a month is less than 31 days, then the missing days are assigned a value of -1.

**Table 2:** Data fields that were prepared for the space-time cube.

Field name	Type	Description
CODE	Numeric	The administrative code of the city
NAME	Text	The name of the city
POPU	Numeric	The population aged 0–9 years in each city
MONTH	Numeric	The month when the HFMD cases were recorded
DAY1-DAY31	Numeric	The count of HFMD cases that were collected from the first day to the last day of one month

The second dataset is used for space-time scan statistics and is organized using Microsoft Excel spreadsheets. The dataset involves seven fields, among which the fields CODE, NAME and POPU have the same meanings as in the first dataset. The field TIME denotes the day when the HFMD data were collected. In accordance with the SaTScan software, the TIME field is presented as “year/month/day”. The fields LONGITUDE and LATITUDE denote the longitude and latitude of the city, respectively.

The last dataset is an ESRI shapefile that stores the city boundaries in Guangdong Province. The most important fields are SHAPE, CODE and NAME. The fields CODE and NAME have the same meanings as in the first dataset. The field SHAPE denotes the administrative boundaries of the 21 prefecture-level cities in Guangdong Province.

The integration of the three datasets is required to build the HFMD database. First, records will be extracted from the first dataset to generate the space-time cube; then, the space-time clusters will be achieved from the SaTScan files; and finally, the aggregation will be displayed on the map.

## Results

### Space-Time Cube of the HFMD incidence in Guangdong Province

**Fig 3:**
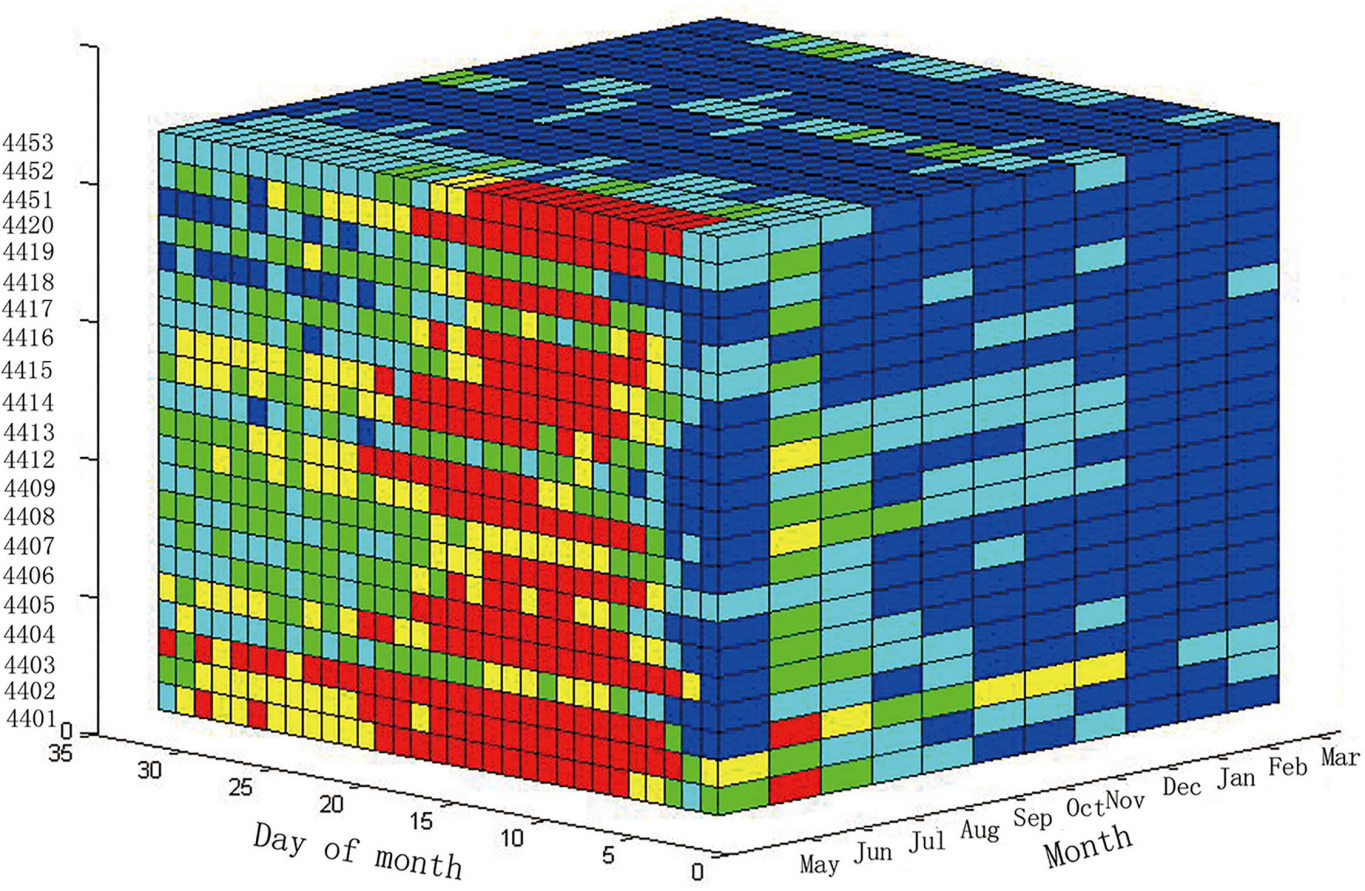
Space-Time Cube of the HFMD incidence in Guangdong Province.

The space-time cube model visualizes the incidence of HFMD in Guangdong Province. To reflect all of the information for HFMD, the target needs to accurately observe the incidence in each city and on each day. There are a total of twenty-one prefecture-level cities; therefore, the number of lines on the vertical axis is twenty-one. In this study, we arranged the vertical axis according to the administrative code. As shown in Fig 3, the horizontal axis to the right represents the months and includes 11 months, from May 2008 to March 2009; the horizontal axis to the left shows the day of the month. To construct an integration cube, every month was assumed to contain 31 days. If the number of days in some months is less than 31 (for example, June has only 30 days), the missing days are filled with black. The total 21 prefecture-level cities are displayed on the vertical axis. The color from blue to red in each grid cell denotes the incidence from low to high, respectively.

The incidence of HFMD in all of the cities during a given period of time could be represented in the STC model. The left plane of the STC (Fig 3) displays the incidence of HFMD in May 2008. As observed from this plane, most grids are red or yellow, indicating the serious incidence of HFMD in May 2008. After slicing the STC along the direction parallel with the left plane, we obtained another plane. This plane displays the HFMD incidence of another month. From the various colors of the grid cells, we can easily decipher the incidence of HFMD.

We could also display the HFMD incidence in one city using the STC model. For example, the upper plane represents the incidence of HFMD in Guangzhou, which is the capital of Guangdong. From this plane, it is easy to observe that there are more blue grids than red grids, indicating that Guangzhou had a mild incidence of HFMD from May 2008 to March 2009.

The STC provides all of the information about the HFMD incidence, and users can obtain the information concerning HFMD for a given period of time or sub-region through the “slice” and “dice” operations. The “slice” operation also facilitates the exploration of the relationship between the incidence of the HFMD and the geographic space or time.

#### Patterns of the HFMD incidence by time

The relationships between the incidence of HFMD and months could be observed using the STC model. This method compares two different planes, which are sliced along the month axis. In this section, we selected two months, May and October, in 2008.

**Fig 4:**
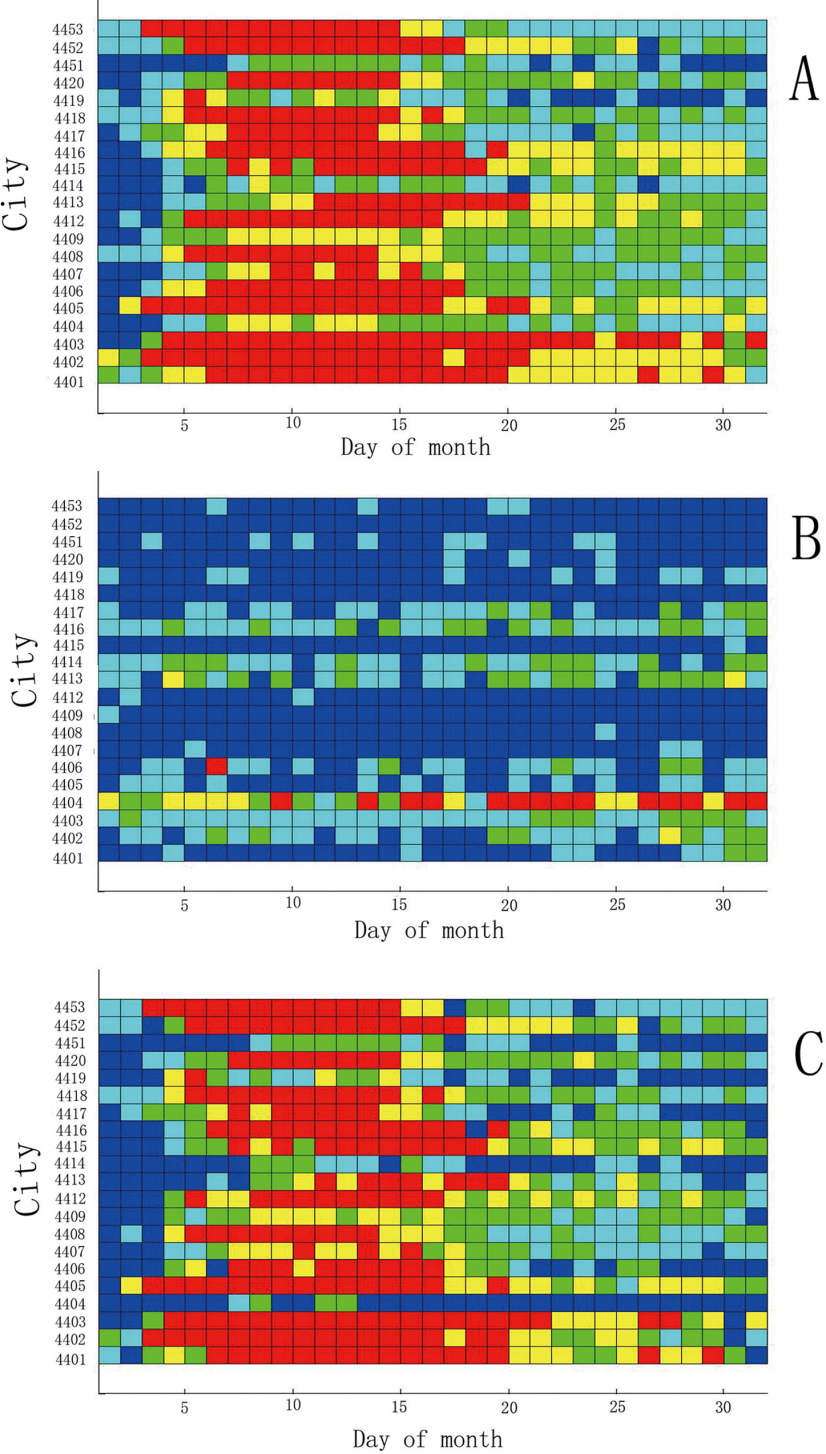
Comparison of the HFMD incidence between May and October 2008 in Guangdong.

Fig 4A shows the HFMD incidence in Guangdong Province in May 2008, where the horizontal axis denotes the days from 1–31, and the vertical axis denotes the cities, with 21 cities in total. Fig 4B presents the same information that is depicted in Fig 4A, except for the month of October. Fig 4C shows the difference in results between May and October.

Some interesting results can be obtained through the comparison operation. Fig 4A indicates the high HFMD incidence in May 2008, as most of the cells are red. From this sub figure, we know that only four cities have red cells before May 5, while others have blue or green, and the HFMD incidence is not serious at this stage. However, the number of red cells increases after May 5. Another 5 cities become red on May 5, and these cities remain red through the next week, indicating that the HFMD incidence is serious across the Guangdong Province. From May 5, most cities become yellow or green, except for Zhuhai, which is a southern city in Guangdong. Throughout May, Zhuhai has the most serious HFMD incidence, as all 25 cells are red. There are no red cells in Jieyang, Shanwei or Shantou. The HFMD incidence was not serious in these cities, which are all located in the southeast of Guangdong. Most of the cities that have red cells, such as Guangzhou and Shenzhen, are located in the south of Guangdong. All of these cities have a strong economy, and the population mobility is relatively high.

As shown in Fig 4B, most of the cells in October 2008 were green, except for Shantou. The HFMD incidence in Shantou remained high for the entire month. There were no blue cells in Shantou, and most of the cells in the last two weeks were red. Fig 4C depicts the difference in incidence between May and October 2008. From the subfigure, we could easily observe that the incidence in May was heavier than in October of 2008. Some other months were also extracted from the STC model (not shown).

Previous studies found that HFMD will massively break out in late spring and early summer [34]. Meteorological factors, including temperature, relative humidity, and rainfall, have a strong influence on the spread of the disease [16,26,34].Because of the rise of the temperature and the increase of precipitation in late spring, the HFMD will spread very quickly. Most previous researches disclosed the discrepancy of the HFMD incidence among seasons through digital data. However, in this study, Fig 4A and 4B clearly displayed the difference between May and October by means of the color of the graphical visualization.

#### Patterns of HFMD incidence by geography

The relationships between the incidence of HFMD and geography can be observed using the STC model. When slicing the STC along the vertical axis, we can visualize the HFMD incidence during eleven months in one city.

**Fig 5:**
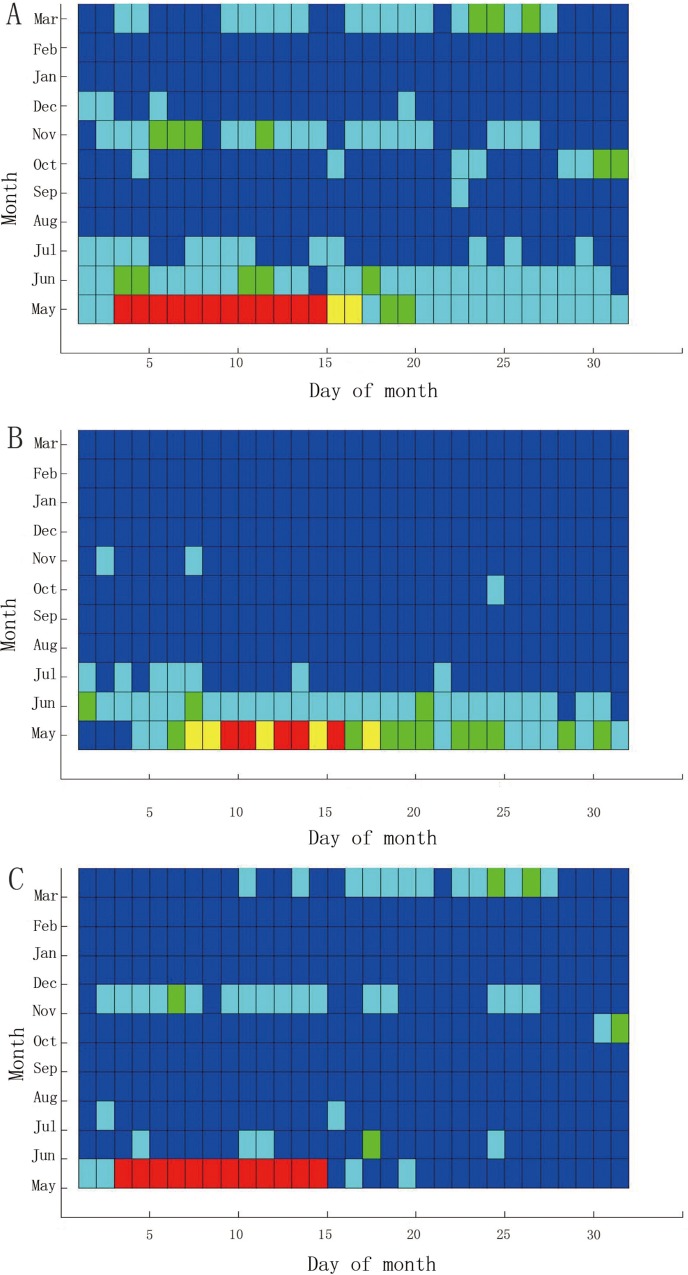
Comparison of incidence of HFMD between Guangzhou and Zhanjiang from May 2008 to March 2009.

Fig 5 shows the comparison of HFMD incidence between Guangzhou and Zhanjiang from May 2008 to March 2009. As shown in Fig 5A and 5C, the HFMD outbreaks primarily occurred in May and June 2008, indicating that HFMD typically occurs in the spring and summer. However, Guangzhou experienced a serious HFMD incidence in November 2008 and March 2009, as the green cells show, perhaps reflecting the large population density and rapid population mobility of Guangzhou. Fig 5C displays the difference between the two cities, showing that the HFMD incidence was more serious in Guangzhou than that in Zhanjiang.

Previous studies found that socio-economic factors have a strong influence on the spread of HFMD incidence [16]. Among the socio-economic factors, the population density, population mobility and industrial structure have a serious impact [34]. In this study, the HFMD incidence was more serious in Guangzhou than that in Zhanjiang by means of graphical visualization. Some possible explanations are as follows: (1) Guangzhou is the capital of Guangdong province, while Zhanjiang is a relatively remote city. The population density and mobility in Guangzhou is more than that in Zhanjiang. This provides the population conditions for the spread of HFMD. (2) The tertiary industry occupied a larger proportion in Guangzhou, while the first industry was largest in Zhanjiang. Through the STC model, we found the HFMD incidence concentrated in the Pearl River Delta region, including Guangzhou, Shenzhen, Foshan and Dongguan. The tertiary industry was activity in these regions, and this provided a favorable condition for the spread of HFMD.

#### Patterns of HFMD incidence by environmental factors

**Fig 6:**
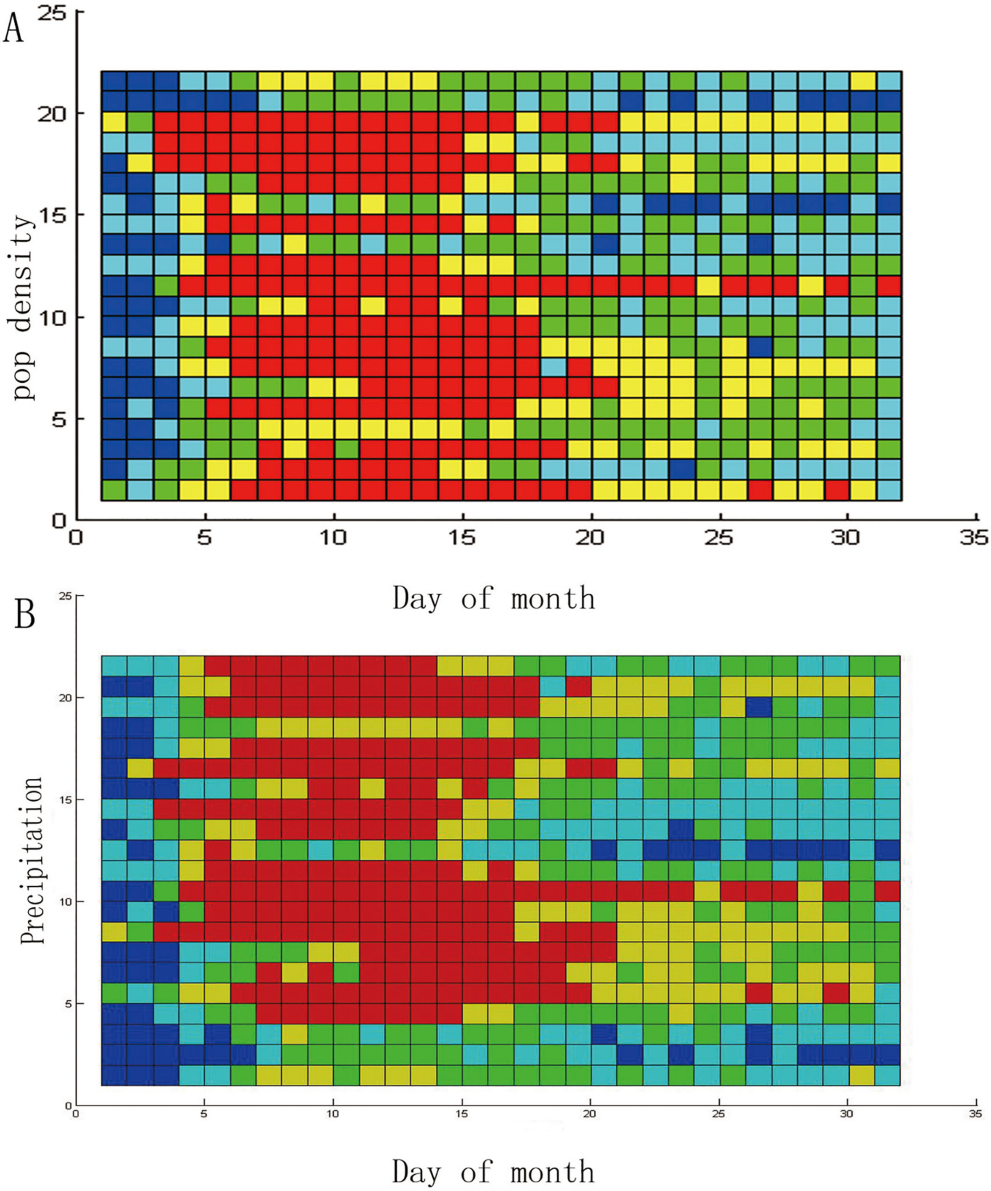
Patterns of HFMD incidence by environmental determinant.

The STC model not only compares discrepancies in time and space but also observes the determinants of the incidence of HFMD. When the order of the vertical axis is arranged in accordance with the order of a determinant, the STC model can be used to observe the influence of the factor on the incidence of HFMD. We have conducted many experiments, including socio-economic and meteorological studies, and observed some phenomena between these factors and the incidence of HFMD. The order of the vertical axis in Fig 6A is arranged according to the size of the population density. As this picture shows, when the population density is relatively small, the outbreak of HFMD (the red units) occurs relatively late, and when the population density gradually increases, the outbreak of HFMD occurs earlier. This phenomenon might reflect a pattern between population density and the incidence of HFMD.

The order of the vertical axis in Fig 6B is arranged according to the amount of precipitation. As this picture shows, the incidence of HFMD is not serious in the three cities where the monthly precipitation is the least. When the precipitation increases, the number of red cells increases, particularly when the precipitation is within a certain range. This phenomenon might indicate that precipitation is related to the incidence of HFMD.

In this research, the association between the HFMD incidence with season, geography and rainfall was discovered through graphical visualization. Because the STC model does not assume that the disease data should obey some statistical distribution, avoiding the error that is caused by an a priori assumption. A further analysis could be conducted by means of the result of the STC model.

### Space-time scan statistics of HFMD’s incidence in Guangdong Province

In the present study, we used a circle as the scanning window for the geographic space. The population of a cylinder is less than 10% of the total population, and the disease case model is a discrete Poisson model. The scanning results are shown in Table 3:

**Table 3:** The results of space-time scan statistics.

Clusters	Regions	Time	No.Cases	No.Expected	LLR	P-Value
1	Shenzhen	2008/5/3–5/16	4683	196.37	10,517.9	1.00E-17
2	Foshan	2008/5/3–5/16	3071	169.56	6056.72	1.00E-17
3	Dongguan	2008/5/4–5/17	1866	173.68	2759.46	1.00E-17
4	Shaoguan, Qingyuan, Heyuan	2008/5/4–7/10	4555	1300.39	2535.94	1.00E-17
5	Maoming, Yangjiang	2008/5/4–5/17	2022	253.75	2451.65	1.00E-17

**Fig 7:**
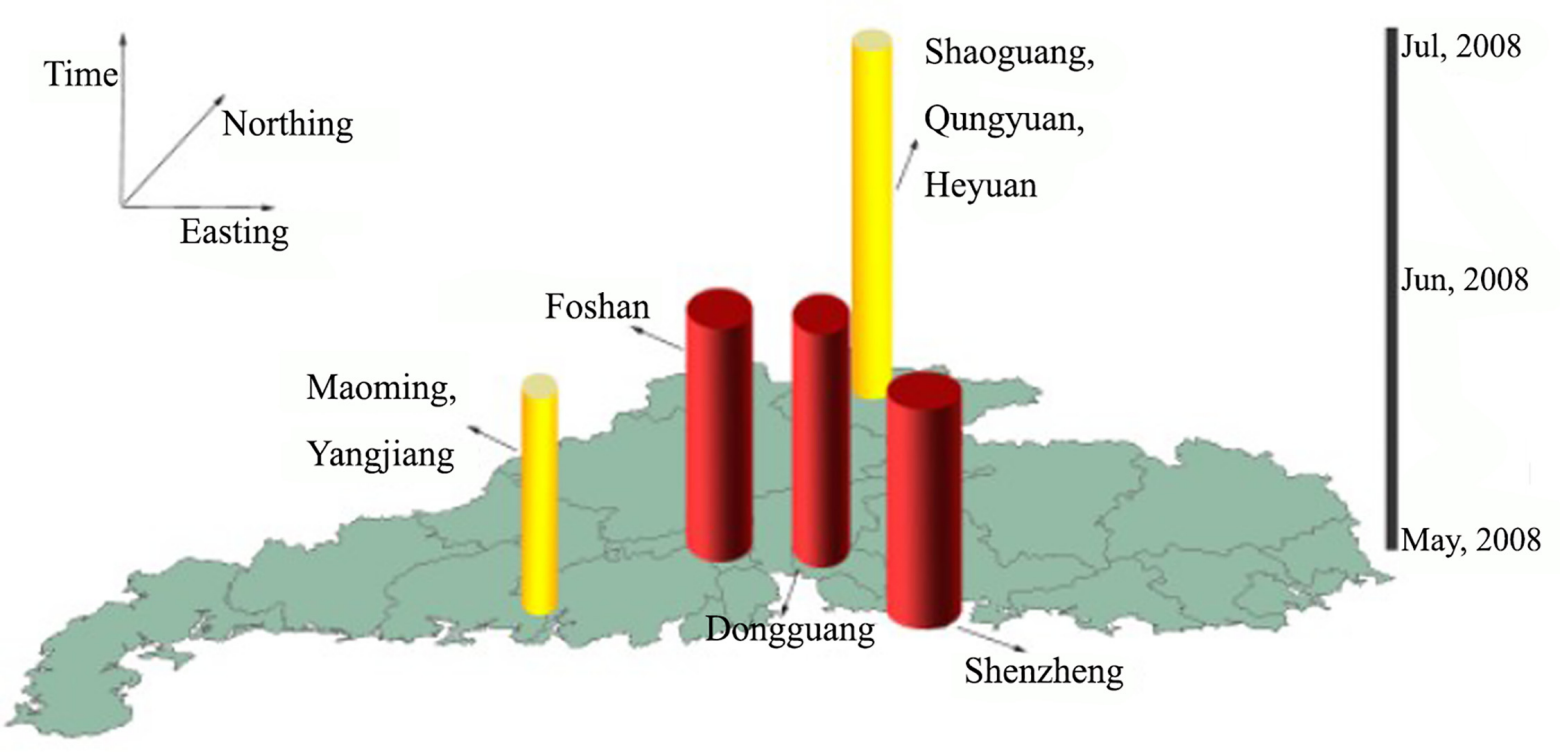
Space-time representations of clusters as detected by space-time scan statistics.

To effectively visualize the results of the space-time scan statistics, we used colorful cylinders to display these aggregations (Fig 7). In Fig 7, the deep red cylinders denote the first three high aggregations, and the yellow cylinders represent the fourth and fifth high aggregations. The cylinders are located in clusters, where the circle of the base of the cylinder is proportional to the largest likely ratio (LLR), the height of the cylinder denotes the time period of the clusters, and the base of the cylinder denotes the geographical space of the HFMD outbreak.

As shown in the Fig 7, the most likely cluster is Shenzhen, and the cluster time is from May 3 to May 16, 2008. The largest likelihood ratio is 10517.9, and the p-value equals 1e-17, indicating that the result is highly significant. The second and third most likely clusters are Foshan and Dongguan, respectively, both with high significance. These three aggregations are located in the middle of Guangdong Province, representing the most economically developed areas of Guangdong. The population density and mobility are high. These factors might reflect the high degree of aggregation of the incidence of Hand- foot-mouth disease.

Regarding time, all of the clusters occurred in May 2008, lasting for half of the month, while some periods lasted until July (e.g., Shaoguan), when the temperature gradually increased and an increased amount of precipitation was observed.

## Discussion

This article applied a method combining the spatiotemporal cube with space-time scan statistics to explore the incidence of HFMD from May 2008 to March 2009 in Guangdong Province. Through the STC model and STSS model, we found that high HFMD incidences were located in the most economically developed areas of Guangdong, and serious HFMD incidence were focused in May 2008, especially in the first half of the month.

Using the STC, the HFMD incidence across the regions and different times was easily observed. The differences between the geography and seasons were also easily explored through the STC. The STC reflects more detailed information, and these details are difficult to observe through digital data. The space-time scan statistics accurately calculated the maximum likely cluster and tested the cluster significance through a Monte Carlo simulation. This article used the STSS model to detect HFMD in Guangdong Province and found that the maximum possible aggregations were located in Shenzhen, Foshan and Dongguan. These areas are located in the southern coastal area of ​​Guangdong Province and have the most developed economies, with large population densities.

Previous studies have shown that population density have a strong influence on the spread of HFMD [22, 23, 26, 34, 35]. Cao et al. found that urban areas have a higher incidence of HFMD than rural areas, and cities with a higher population density and stronger population mobility suffer more from HFMD [36]. Huang et al. found that tertiary industry had a greater impact on HFMD than first industry. This research further confirmed that serious HFMD incidence was located in the most economically developed areas of Guangdong [34]. To prevent a large-scale outbreak of hand, foot and mouth disease, it is necessary to monitor the HFMD incidence in these regions.

We found that high HFMD incidences were focused in May 2008, especially in the first half of the month. Previous studies have shown a clear seasonal pattern for HFMD outbreaks, with a peak in late spring and early summer [16,21,37]. Compared with the previous results, on the one hand, we confirmed the seasonal pattern of HFMD incidence through the visualization analysis; on the other hand, it is easy to detect HFMD incidence across all cities at the same time through the STC analysis model. Our study also showed that precipitation has a certain effect on the incidence of HFMD. Hii et al. found that precipitation has a 1–2-week lag effect on the incidence of HFMD [38]. Wang et al. used the S-BME spatial-temporal model and found that there is a strong relationship between the incidence of HFMD and monthly rainfall [16]. Compared with the previous research, we described the relationship between HFMD incidence with rainfall through visualization, and this result was easy to understand.

Both the STC and STSS reflect the spatiotemporal clusters of this epidemic, but there are some differences between them. Because the STC only provides relatively rough clusters, which rely on the colors of the plane or grids, and because the STSS uses the maximum likelihood ratio statistical technique, precise aggregations can be calculated. Generally, it is better to obtain information for precise aggregations; however, information from other regions is ignored in this process.

The STC has some distinguishing features. First, the STC displays comprehensive information concerning the epidemic. The STC is a three-dimensional visual tool, through which the information for any location or time can easily be displayed. In addition, the STC provides many functions that have been previously illustrated. These functions can solve many spatiotemporal problems, such as the relationship of the epidemic incidence between two months. Thus, the STC is not only a visualization tool but also a powerful analytical tool. Moreover, the STC does not assume that the disease data should obey some statistical distribution, thereby avoiding the error that is caused by an a priori assumption.

The STSS also has distinguishing features. The most important feature of STSS is accuracy, as this method uses the maximum likelihood ratio statistical method. Because the maximum likelihood ratio reflects the information both inside and outside of the clusters, the probability of the occurrence of the disease can objectively be explained. Due to the distinctive features of these two models, it is necessary to analyze the disease data in stages. First, we constructed a database of disease data, and an STC model was constructed to observe the overview of the disease distribution. Subsequently, the STSS model was used to precisely calculate aggregations.

There are some shortcomings of STC, among which color grading is the biggest deficiency. Different colors greatly influence the visual effects. Different colors are assigned to some data, which only display small differences. Even using color, it is difficult to visualize the actual differences between these data. Classifying the data to obtain a better visual effect is the next step in developing future applications with this technology. Another area of concern is the arrangement on the vertical axis. The regions in geographic area have spatial autocorrelation; when they are projected on the vertical axis, these attributes are lost. Thus, the arrangement of these geographic units is also an important study target.

## Conclusion

This article applied a method combining the spatiotemporal cube with space-time scan statistics to explore the incidence of HFMD from May 2008 to March 2009 in Guangdong Province. Through the STC model, we observed that high HFMD incidences were focused in May 2008. Most of the cities that had serious HFMD incidence are located in the south of Guangdong. All of these cities have strong economies. We also found that the HFMD incidence varied among different months and cities. Using the STSS model, we found that the most likely cluster was Shenzhen, and the second and third most likely clusters were Foshan and Dongguan, respectively. All of these three cities are the most economically developed areas of Guangdong.

## Supporting Information

S1 Dataset All data used in tables and figures for this study. (XLSX)
